# Supplementary figures and images for: Transcriptome-wide analysis of the differences between MCF7 cells cultured in DMEM or αMEM
Source: PLoS One. 2024 Mar 28;19(3):e0298262. doi: 10.1371/journal.pone.0298262 (PMC10977736; doi:10.1371/journal.pone.0298262)

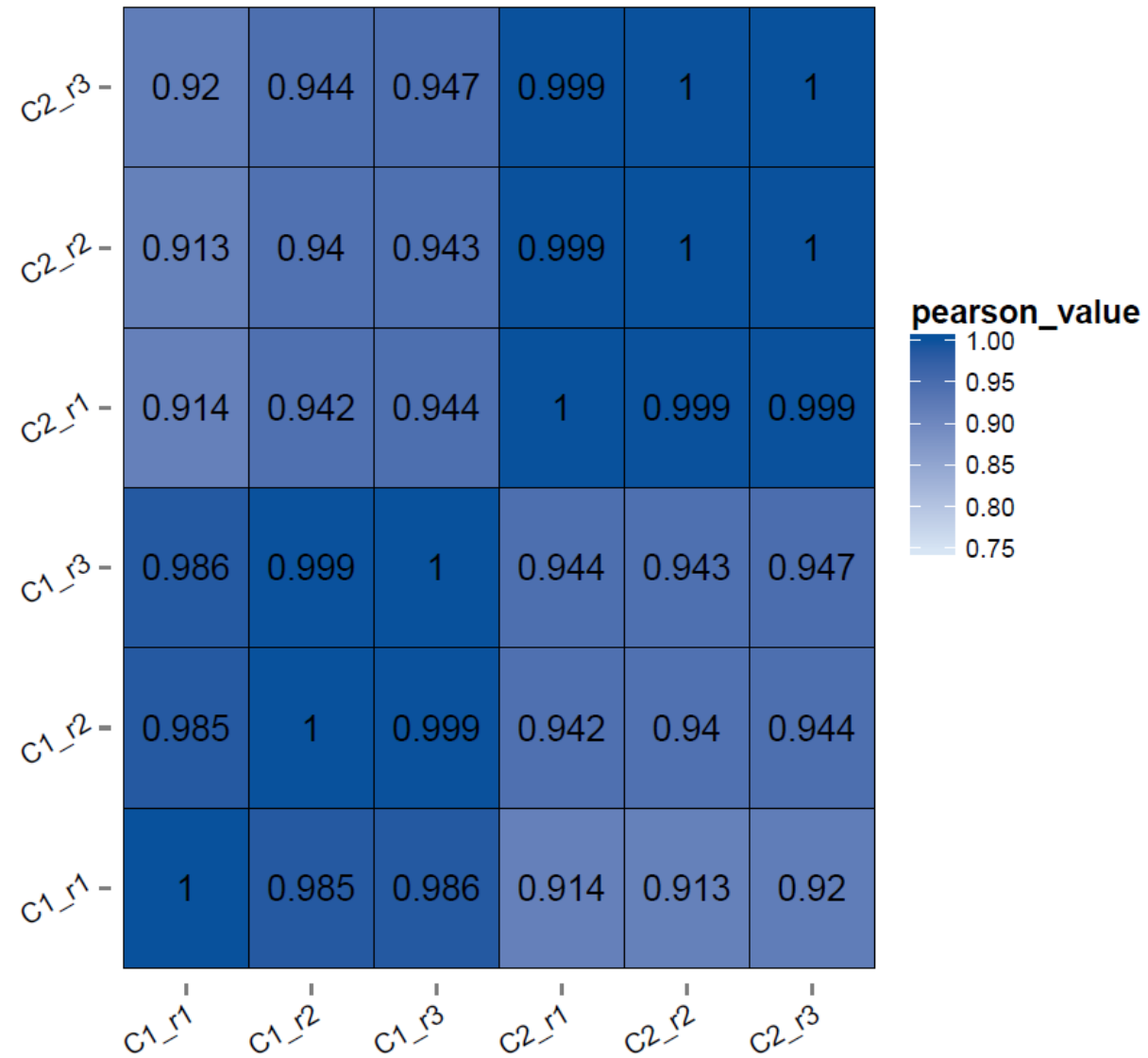

S1 Fig. Correlation analysis between samples

Supplement: S1 Fig — The X and Y axis represent each sample. The color represents the correlation coefficient (the darker the color, the higher the correlation, the lighter the color, the lower the correlation). (PDF) [file pone.0298262.s001.pdf]

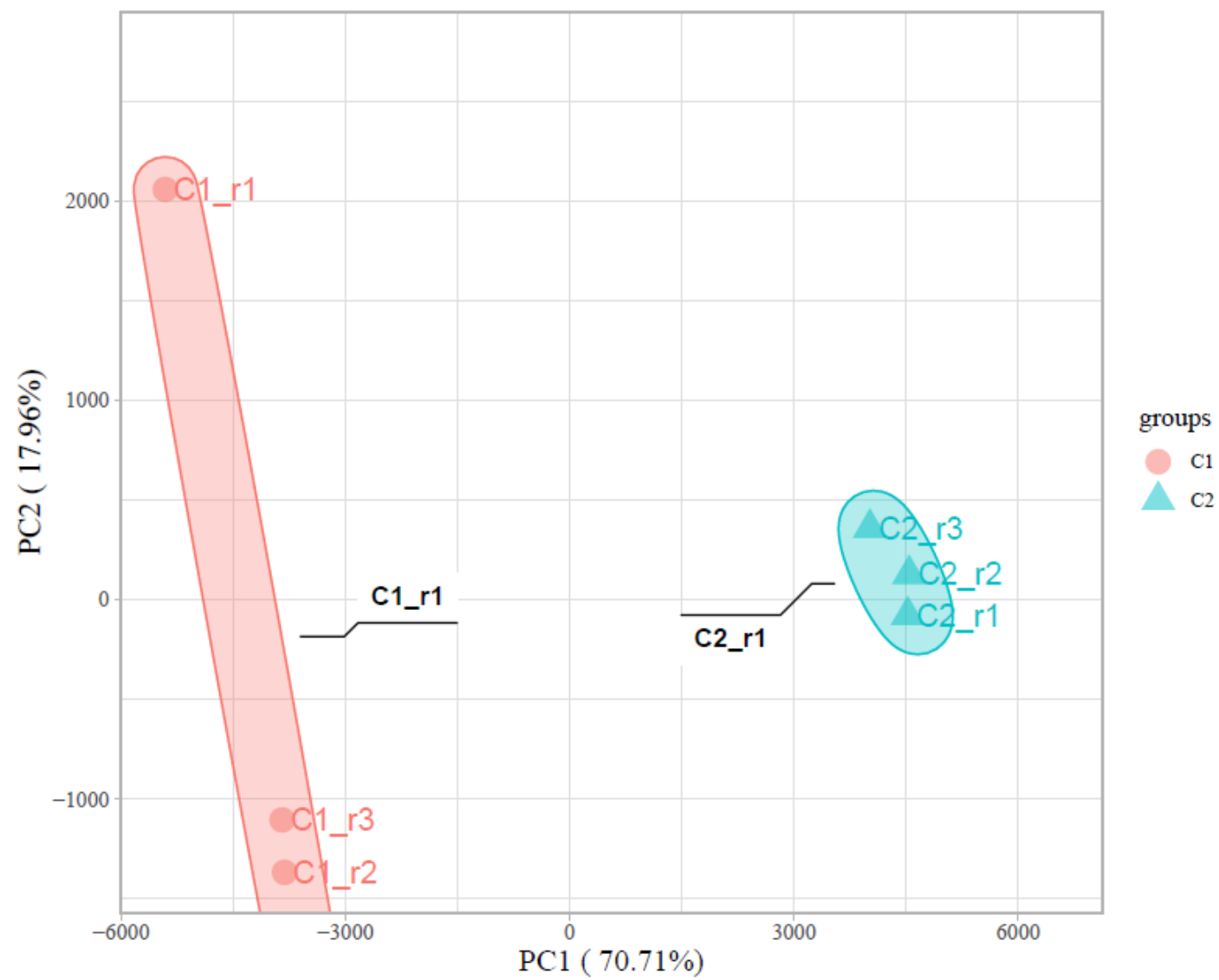

S2 Fig. PCA analysis

Supplement: S2 Fig — X axis represents the contributor rate of first component. Y axis represents the contributor rate of second component. Points represent each sample. The samples in one group shows the same color. (PDF) [file pone.0298262.s002.pdf]
