# Supplementary material for: Transcriptome-wide analysis of the differences between MCF7 cells cultured in DMEM or αMEM
Source: PLoS One. 2024 Mar 28;19(3):e0298262. doi: 10.1371/journal.pone.0298262 (PMC10977736; doi:10.1371/journal.pone.0298262)
Supplement: S1 Table — Total Raw Reads(Mb): The reads amount before filtering. Unit: Mb. Total Clean Reads(Mb): The reads amount after filtering. Unit: Mb. Total Clean Bases(Gb): The total base amount after filtering. Unit: Gb. Clean Reads Q20(%): The Q20 value for the clean reads. Clean Reads Q30(%): The Q20 value for the clean reads. Clean Reads Ratio(%): The ratio of the amount of clean reads. (PDF) [file pone.0298262.s003.pdf]

S Table. Clean reads quality metrics

Sample	Total Raw Reads(Mb)	Total Clean Reads(Mb)	Total Clean Bases(Gb)	Clean Reads Q20(%)	Clean Reads Q30(%)	Clean Reads Ratio(%)
**C1_r1**	23.97	23.93	1.20	98.60	92.32	99.83
**C1_r2**	24.00	23.96	1.20	98.80	93.09	99.83
**C1_r3**	24.00	23.96	1.20	98.77	92.95	99.85
**C2_r1**	23.97	23.92	1.20	98.61	92.46	99.80
**C2_r2**	23.98	23.91	1.20	98.77	92.98	99.73
**C2_r3**	23.99	23.95	1.20	98.61	92.38	99.82
