# Supplementary material for: Transcriptome-wide analysis of the differences between MCF7 cells cultured in DMEM or αMEM
Source: PLoS One. 2024 Mar 28;19(3):e0298262. doi: 10.1371/journal.pone.0298262 (PMC10977736; doi:10.1371/journal.pone.0298262)
Supplement: S1 Raw images — (PDF) [file pone.0298262.s004.pdf]

P21-A

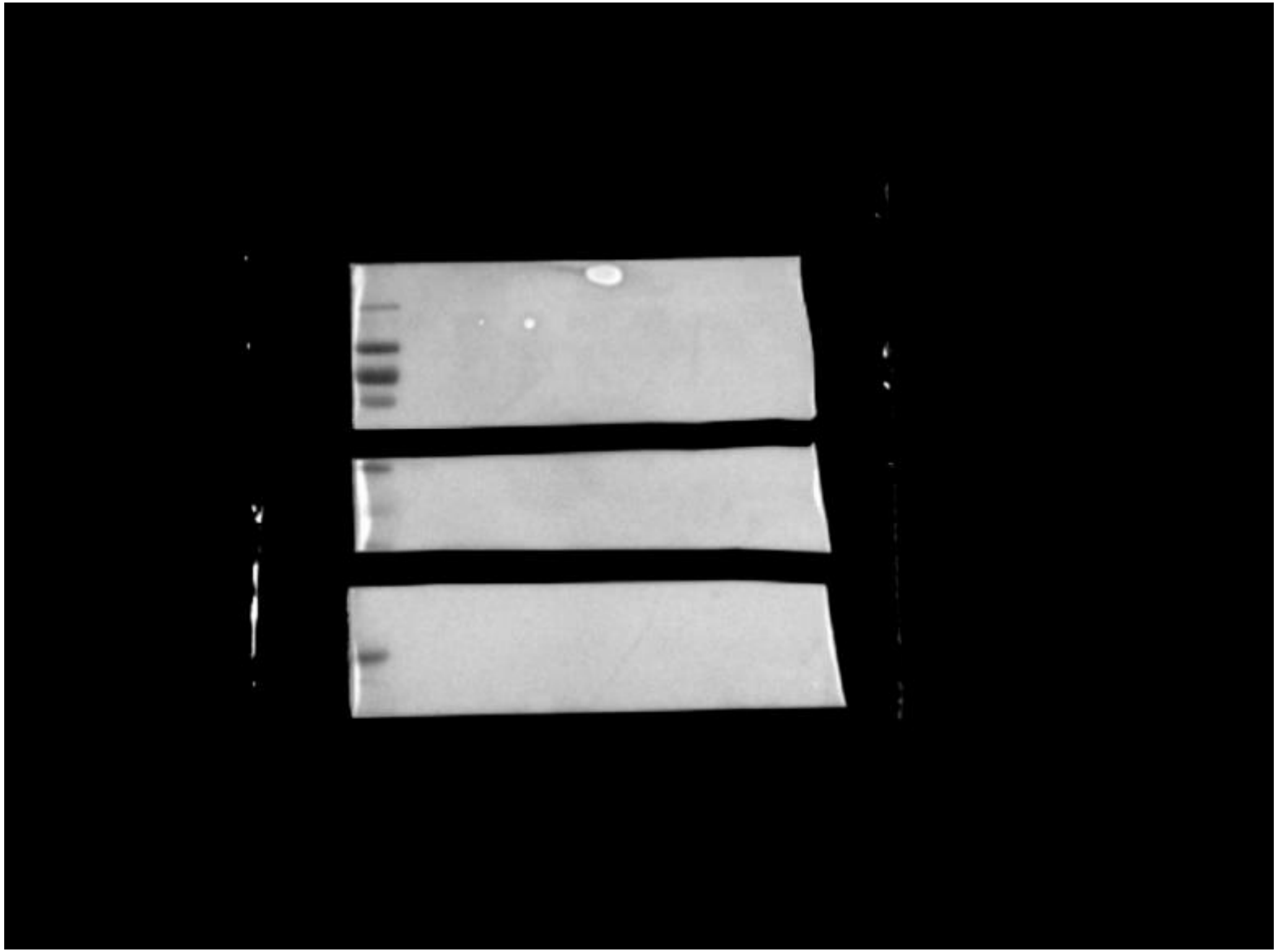

P21-A： This picture was got from BIO-RED gel imaging system (Model No. PowerPac Universal Power Supply) with Image Lab 5.2 system. The Custom-epi protocol was used. The exposure time was 0.5 s.

P21-B

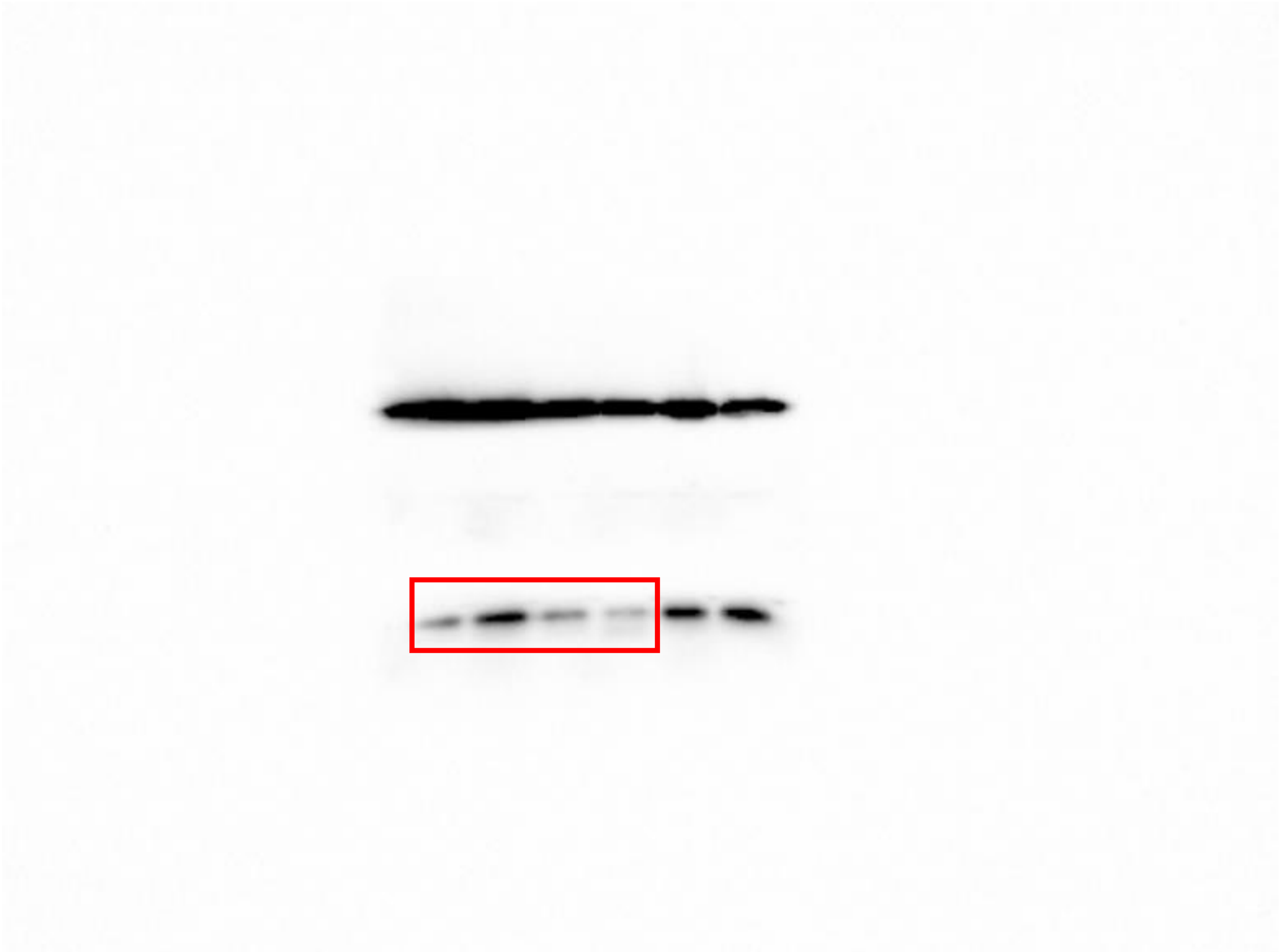

P21-B： This picture was got from BIO-RED gel imaging system (Model No. PowerPac Universal Power Supply) with Image Lab 5.2 system. The Blots-Chemi protocol was used. The exposure time was 1 s. The red box represent the selected bands in the manuscript

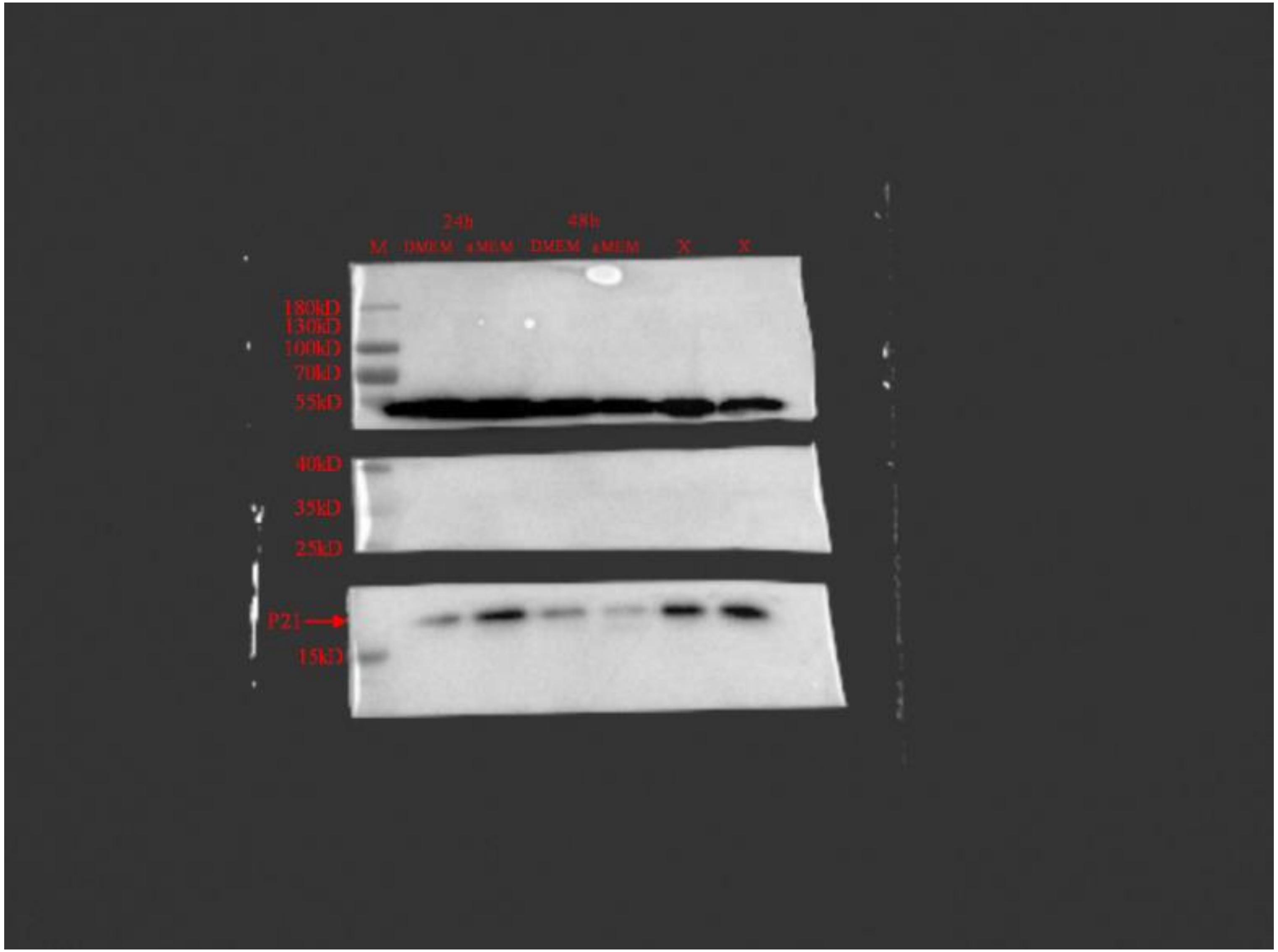

This picture was merged by P21-A and P21-B. Protein ladder and sample names were marked in red font

CDK1-A

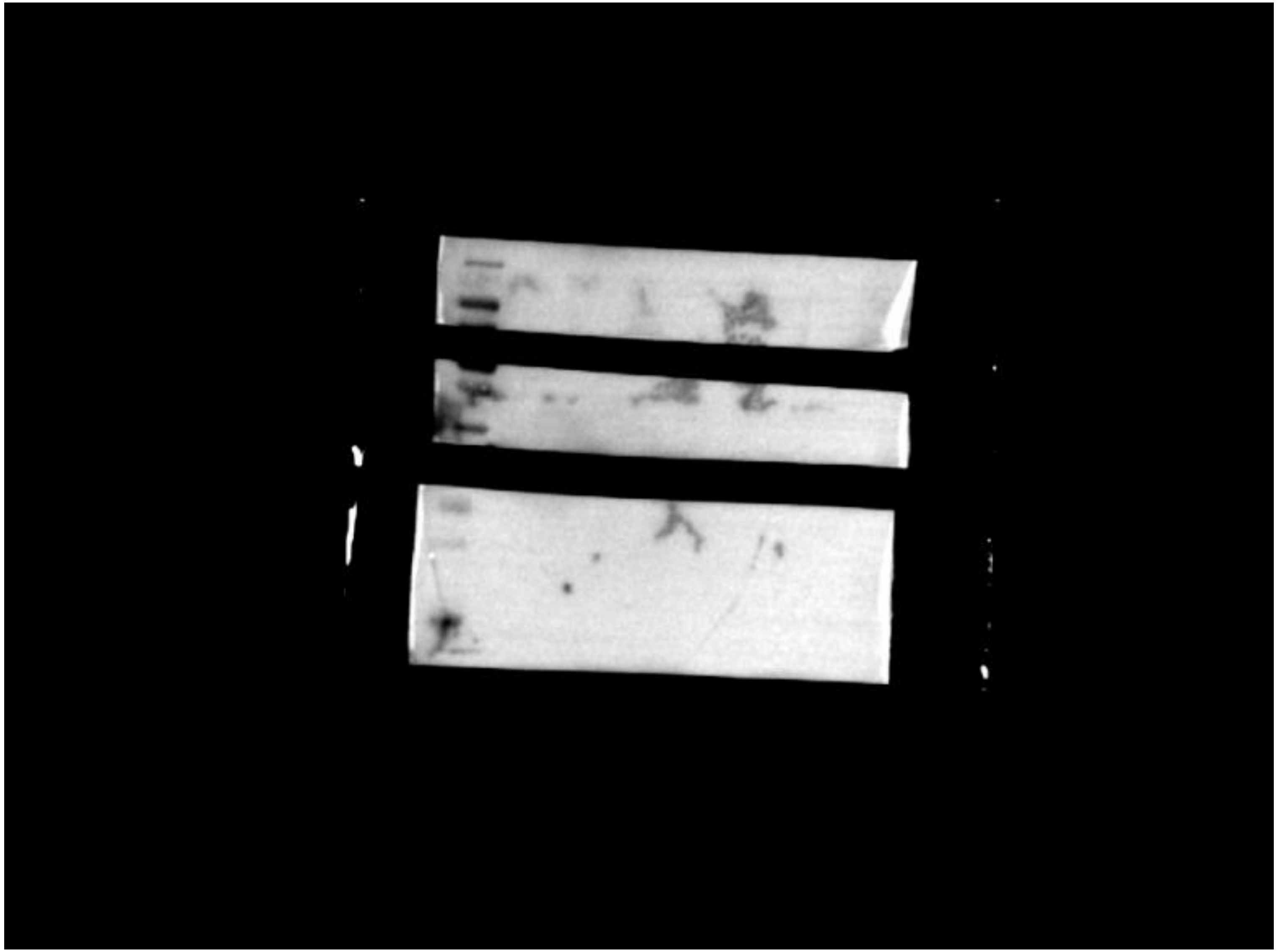

CDK1-A： This picture was got from BIO-RED gel imaging system (Model No. PowerPac Universal Power Supply) with Image Lab 5.2 system. The Custom-epi protocol was used. The exposure time was 0.5 s.

CDK1-B

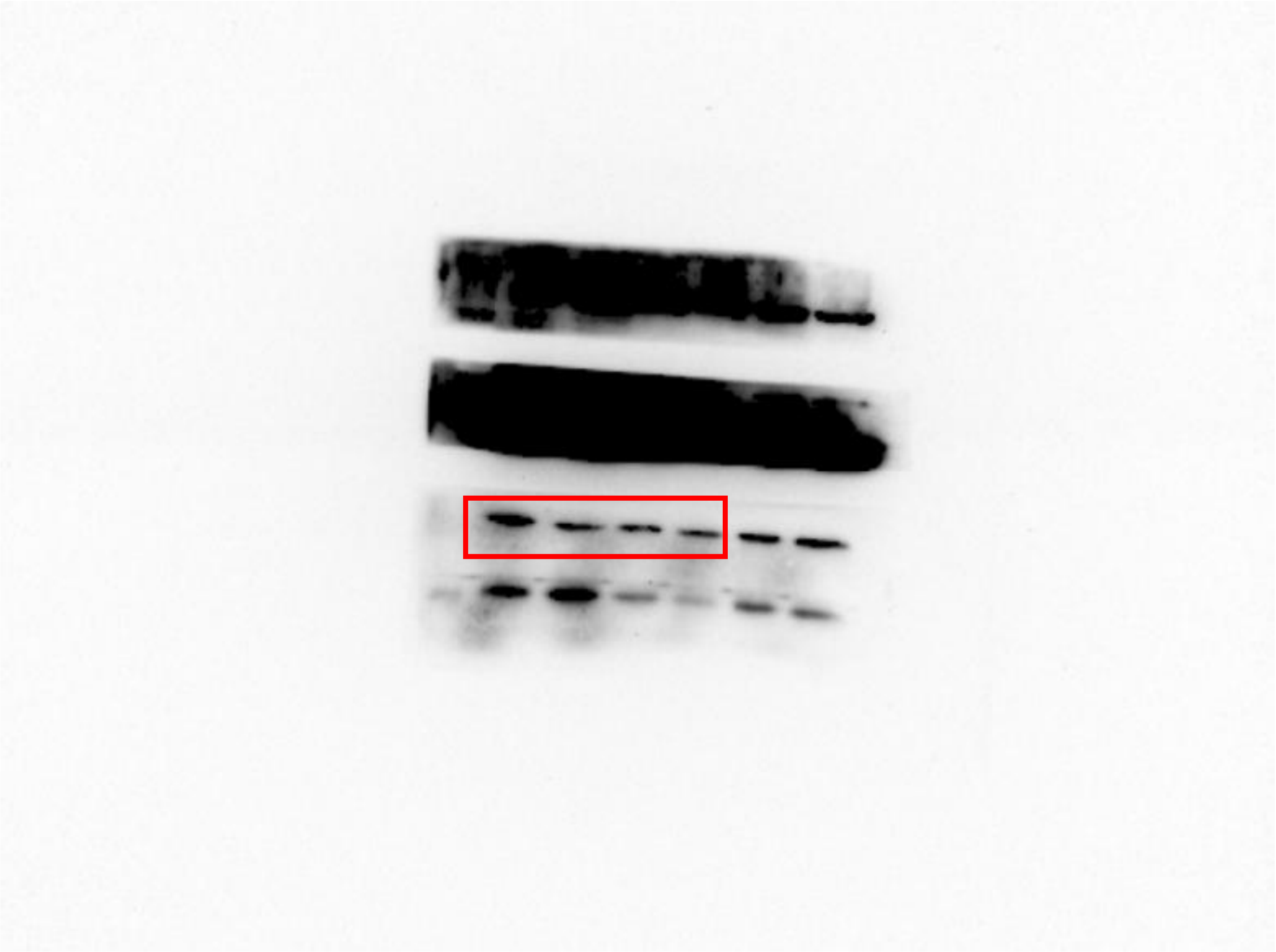

P21-B： This picture was got from BIO-RED gel imaging system (Model No. PowerPac Universal Power Supply) with Image Lab 5.2 system. The Blots-Chemi protocol was used. The exposure time was 15 s. The red box represent the selected bands in the manuscript

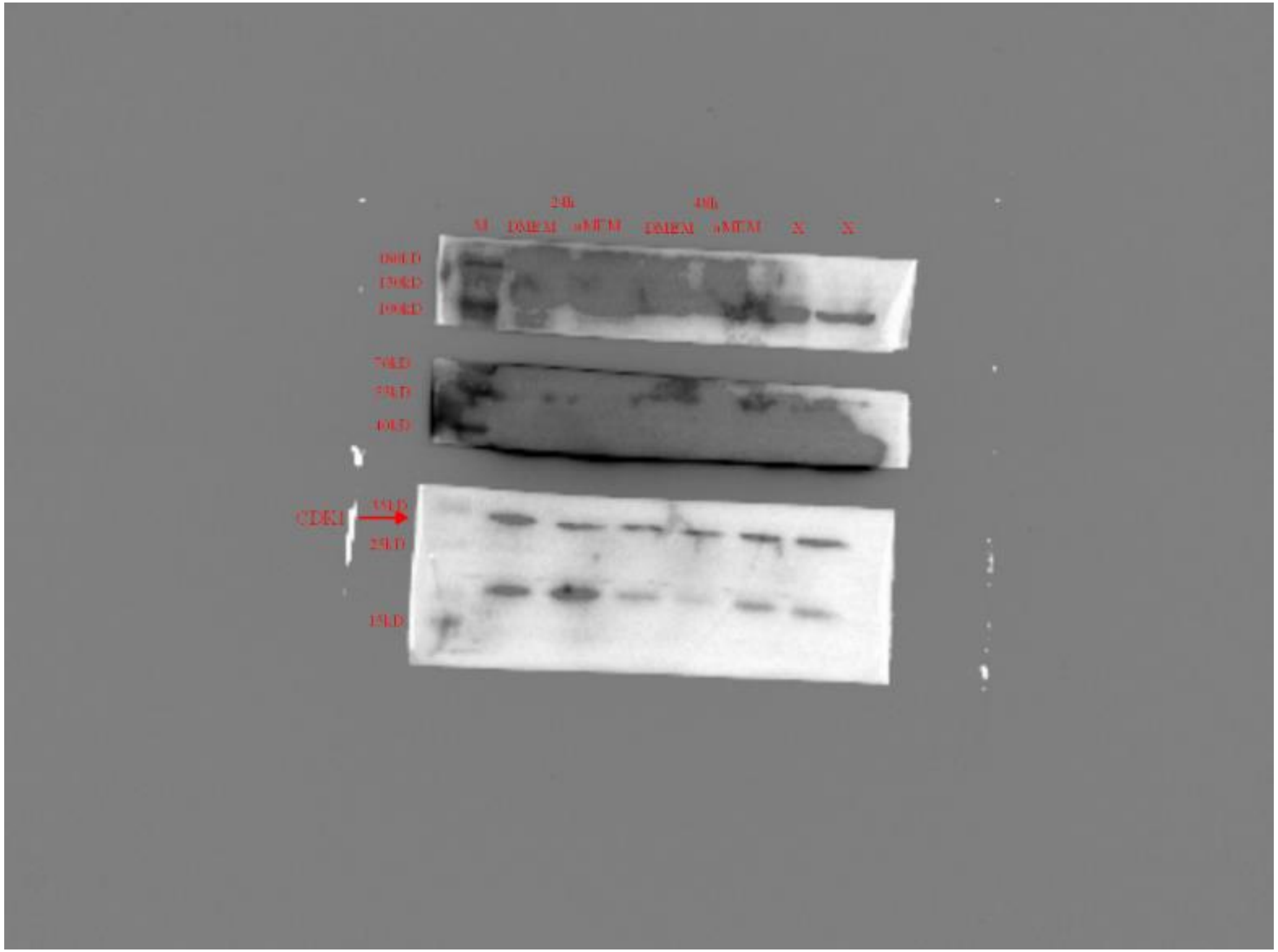

This picture was merged by CDK1-A and CDK1-B. Protein ladder and sample names were marked in red font

GAPDH-A

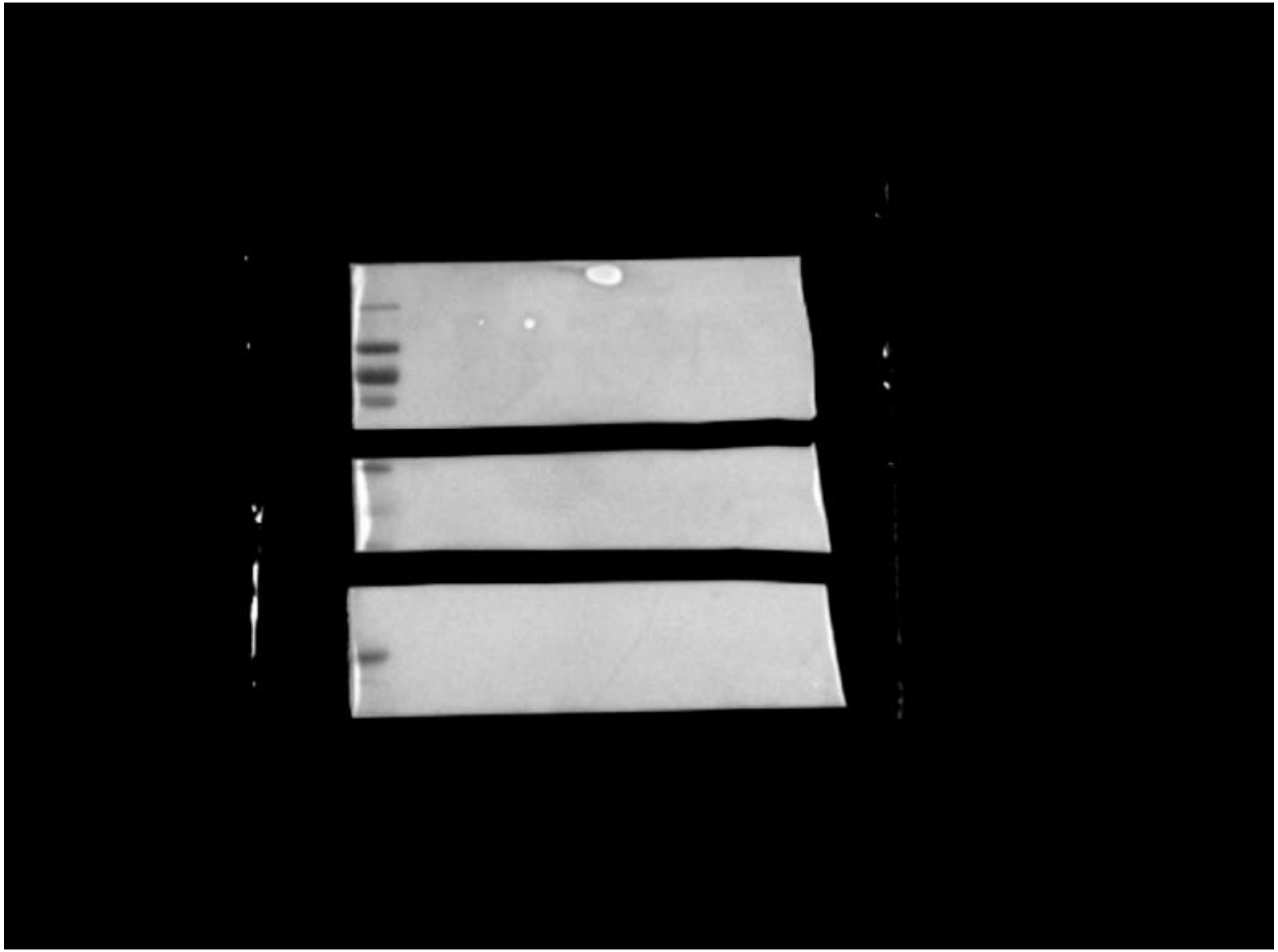

GAPDH-A： This picture was got from BIO-RED gel imaging system (Model No. PowerPac Universal Power Supply) with Image Lab 5.2 system. The Custom-epi protocol was used. The exposure time was 0.5 s.

GAPDH-B

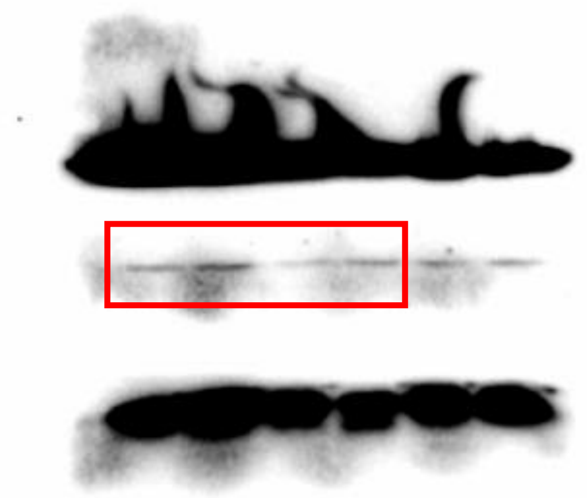

GAPDH-B： This picture was got from BIO-RED gel imaging system (Model No. PowerPac Universal Power Supply) with Image Lab 5.2 system. The Blots-Chemi protocol was used. The exposure time was 10 s. The red box represent the selected bands in the manuscript

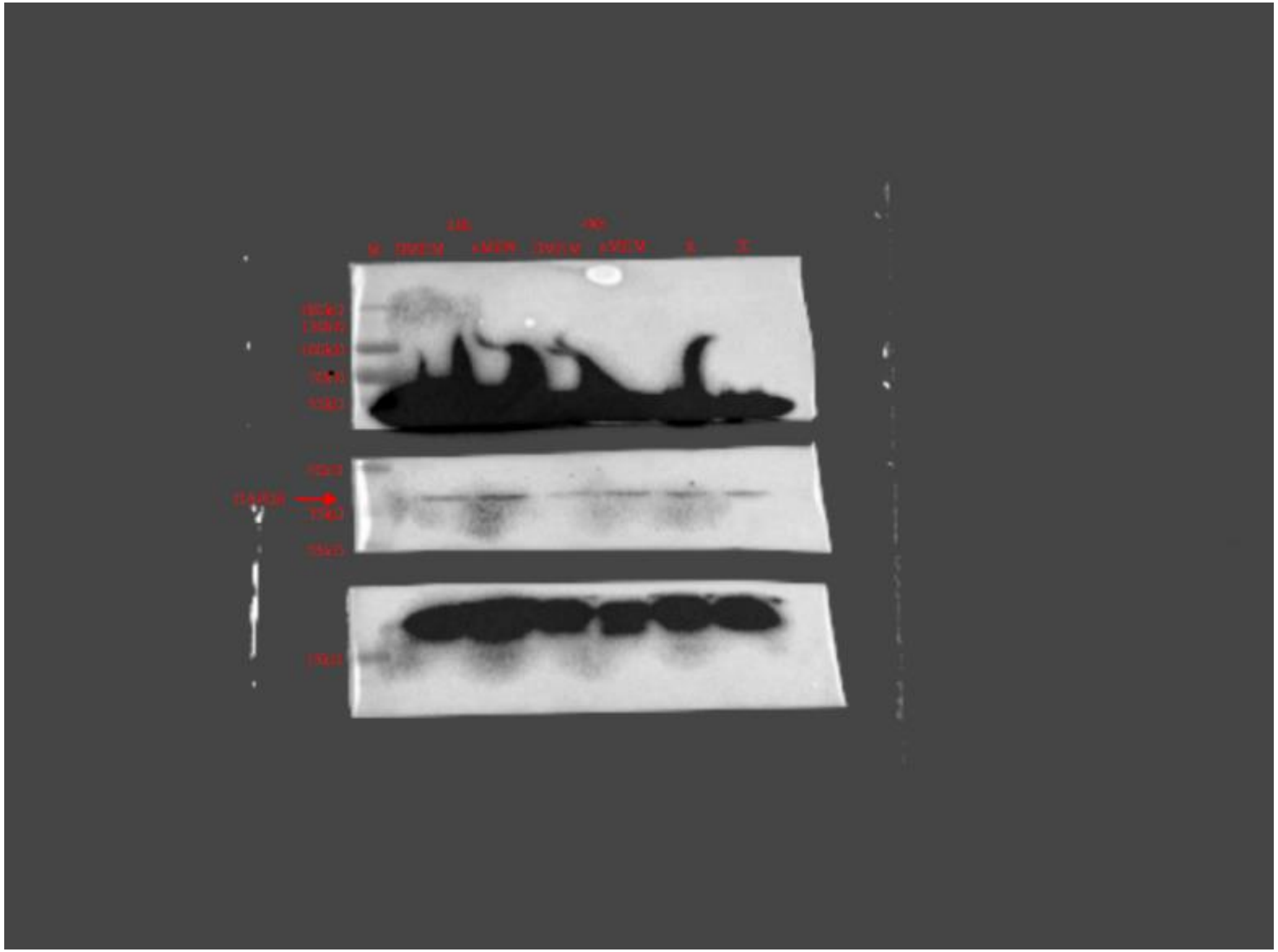

This picture was merged by GAPDH-A and GAPDH-B.
Protein ladder and sample names were marked in red font

β-actin-A

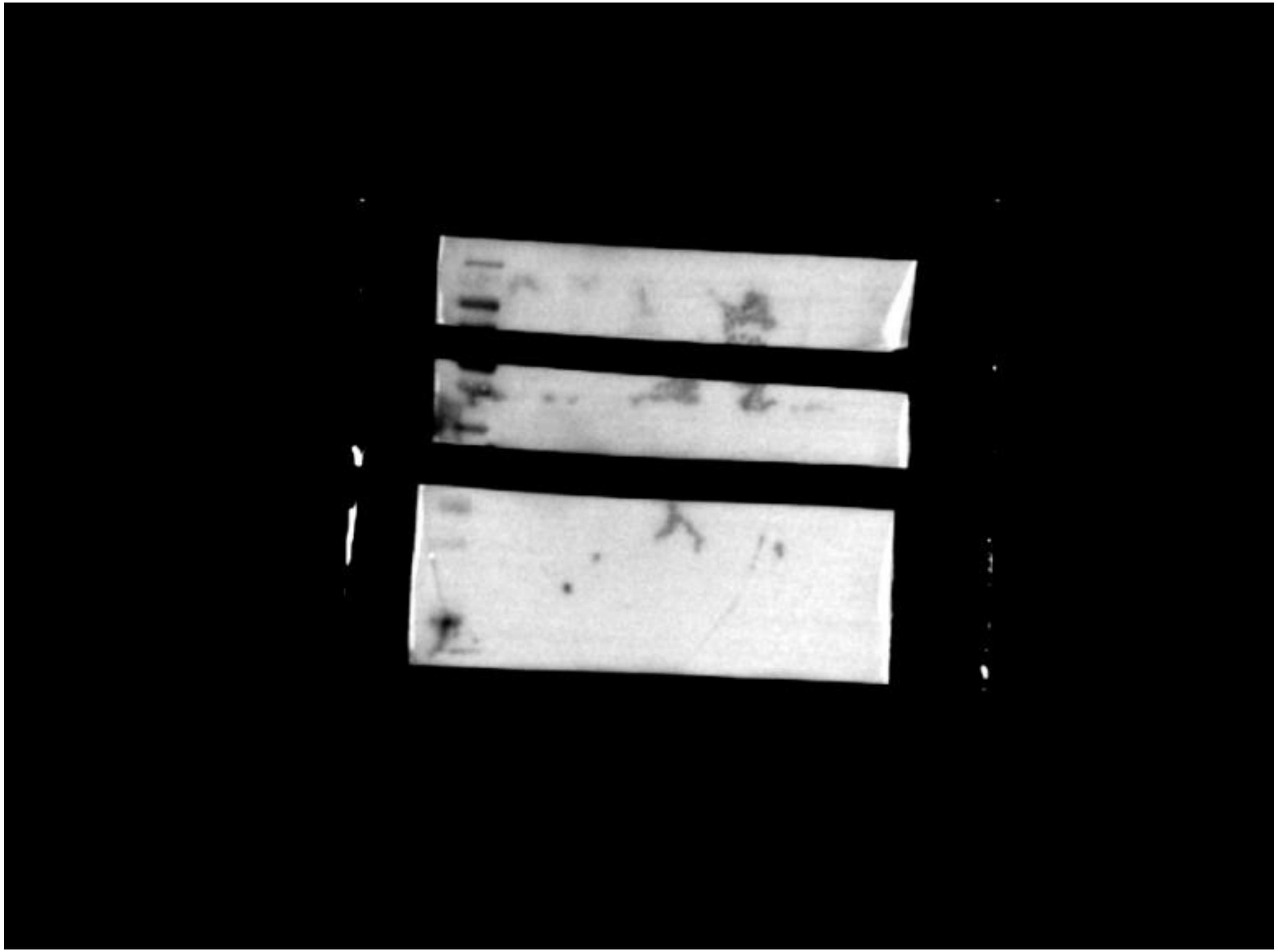

β-actin-A： This picture was got from BIO-RED gel imaging system (Model No. PowerPac Universal Power Supply) with Image Lab 5.2 system. The Custom-epi protocol was used. The exposure time was 0.5 s.

β-actin-B

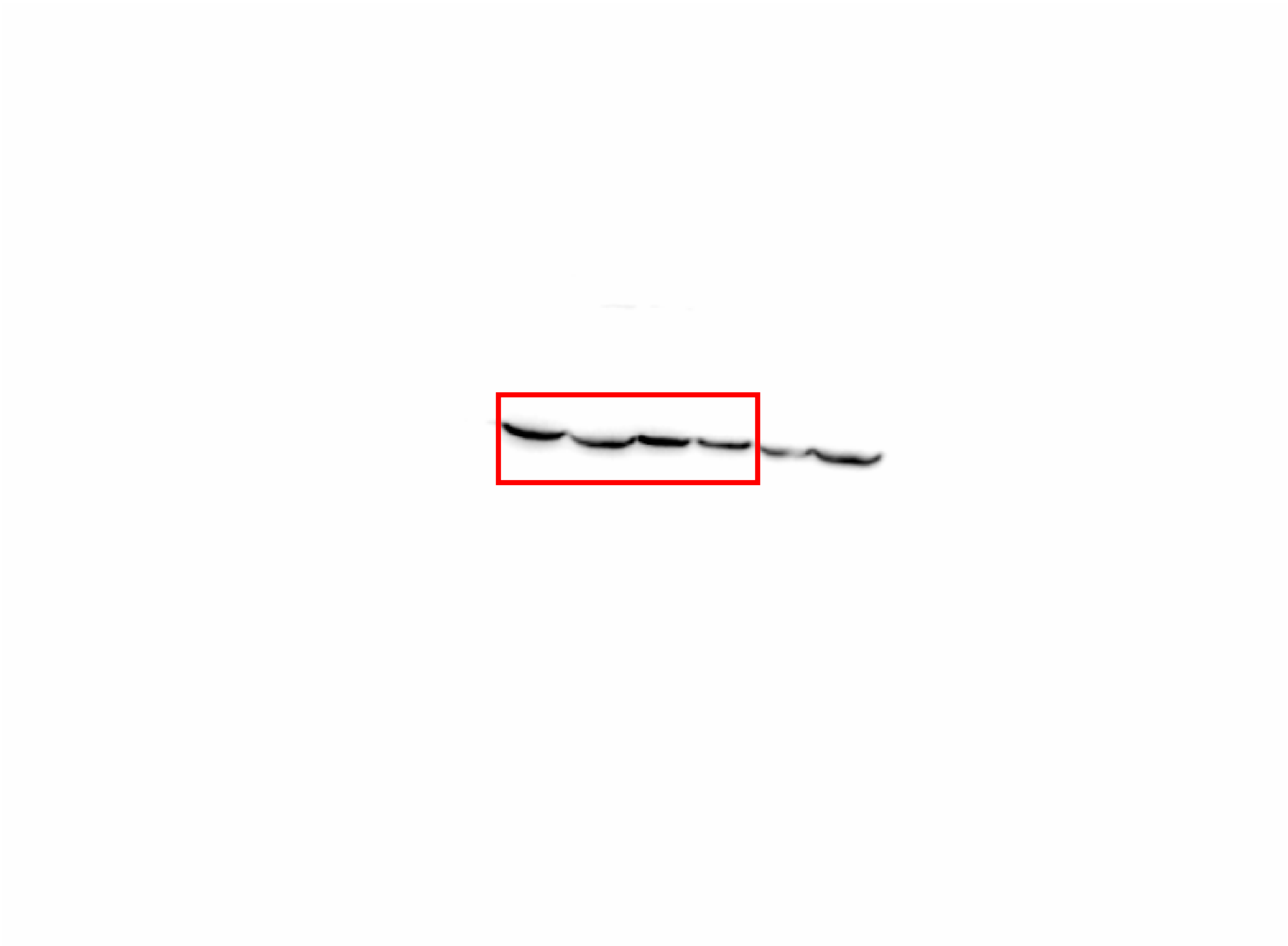

β-actin-B： This picture was got from BIO-RED gel imaging system (Model No. PowerPac Universal Power Supply) with Image Lab 5.2 system. The Blots-Chemi protocol was used. The exposure time was 1 s. The red box represent the selected bands in the manuscript

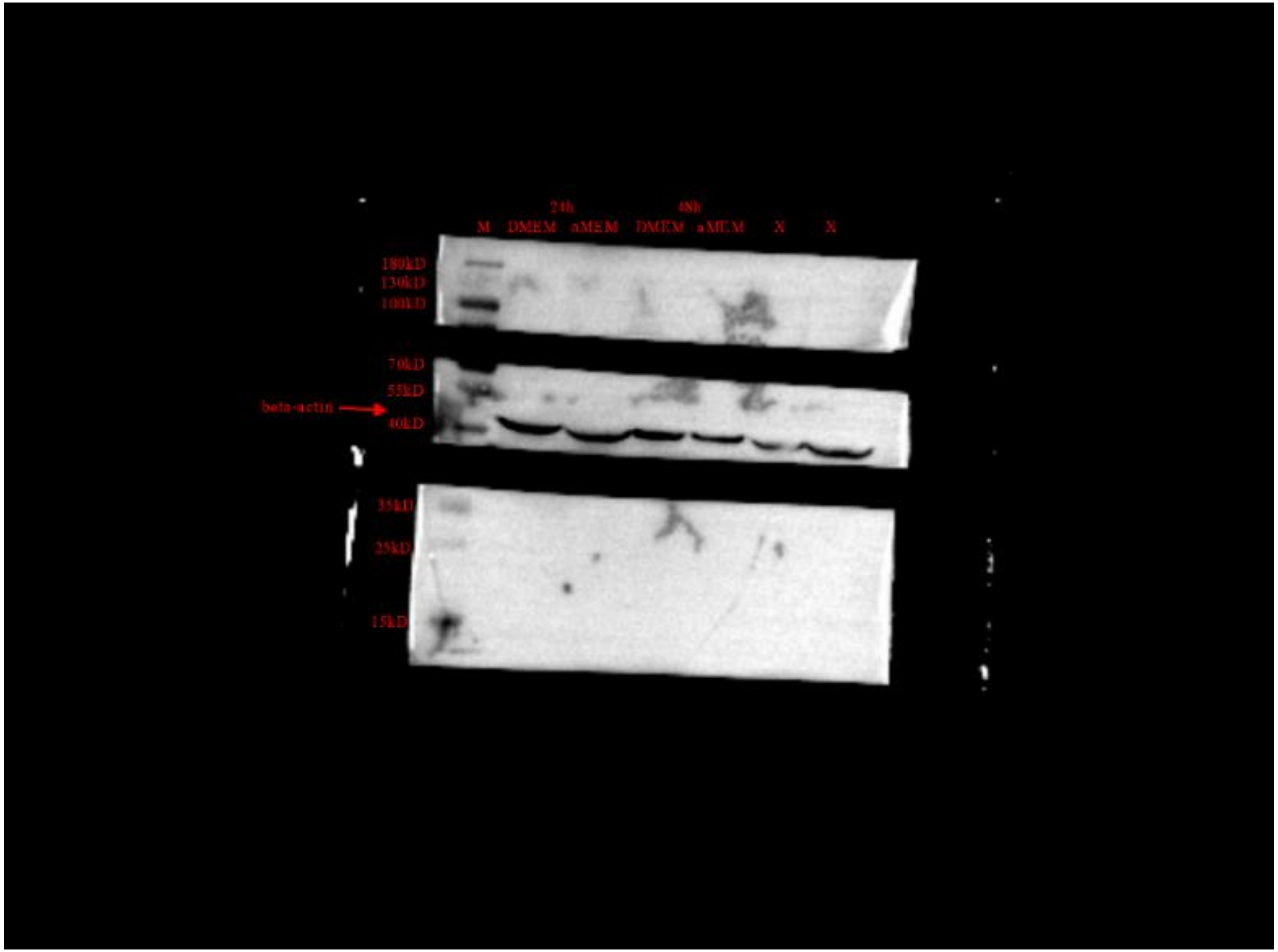

This picture was merged by β-actin-A and β-actin-B. Protein ladder and sample names were marked in red font
