# Supplementary material for: Transcriptome-wide analysis of the differences between MCF7 cells cultured in DMEM or αMEM
Source: PLoS One. 2024 Mar 28;19(3):e0298262. doi: 10.1371/journal.pone.0298262 (PMC10977736; doi:10.1371/journal.pone.0298262)
Supplement: Attachment — Submitted filename: Response to Reviewers.docx [file pone.0298262.s005.docx]

Dear Editor,

Thank you for giving us the opportunity to revise our manuscript entitled “Transcriptome-wide analysis of the difference between MCF7 cells cultured in DMEM or αMEM (Manuscript ID: PONE-D-23-29345)". Sorry for the late submission. We now have completed our revision. The responds to the reviewer’s comments are as flowing:

1. Please ensure that your manuscript meets PLOS ONE's style requirements, including those for file naming. The PLOS ONE style templates can be found at
<https://journals.plos.org/plosone/s/file?id=wjVg/PLOSOne_formatting_sample_main_body.pdf> and <https://journals.plos.org/plosone/s/file?id=ba62/PLOSOne_formatting_sample_title_authors_affiliations.pdf>

We have revised the format of our article according to PLOS ONE's style requirements, including file naming.

2. We noticed you have some minor occurrence of overlapping text with the following previous publication(s), which needs to be addressed: <https://www.hindawi.com/journals/sci/2018/5912194/>

In your revision ensure you cite all your sources (including your own works), and quote or rephrase any duplicated text outside the methods section. Further consideration is dependent on these concerns being addressed.

We have quoted or rephrased the duplicated text outside the methods section.

3. Note from Emily Chenette, Editor in Chief of PLOS ONE, and Iain Hrynaszkiewicz, Director of Open Research Solutions at PLOS: Did you know that depositing data in a repository is associated with up to a 25% citation advantage (<https://doi.org/10.1371/journal.pone.0230416>)? If you’ve not already done so, consider depositing your raw data in a repository to ensure your work is read, appreciated and cited by the largest possible audience. You’ll also earn an Accessible Data icon on your published paper if you deposit your data in any participating repository (<https://plos.org/open-science/open-data/#accessible-data>).

The mRNA-seq data from this study has been deposited in the NCBI sequence read archive under the BioProject number PRJNA779251, with the fq files spanning accession numbers SRR16914480-SRR16914481.

4. We note that the grant information you provided in the ‘Funding Information’ and ‘Financial Disclosure’ sections do not match.

When you resubmit, please ensure that you provide the correct grant numbers for the awards you received for your study in the ‘Funding Information’ section.

We have corrected the ‘Funding Information’ according to the ‘Financial Disclosure’ sections, and we ensure that the grant numbers are correct.

5. Thank you for stating the following financial disclosure:
"This work was supported by Major Project of Yunnan Science and Technology Program [grant number 202002AA100007 and 202102AA100007-3], Scientific Research Foundation of the Education Department of Yunnan Province [grant number 2021J0226], Joint Special Funds for the Department of Science and Technology of Yunnan Province-Kunming Medical University [grant numbers 2019FE001(-176), 202101AY070001-075]."

Please state what role the funders took in the study.  If the funders had no role, please state: "The funders had no role in study design, data collection and analysis, decision to publish, or preparation of the manuscript."
If this statement is not correct you must amend it as needed.
Please include this amended Role of Funder statement in your cover letter; we will change the online submission form on your behalf.

We have corrected the ‘Funding Information’ according to the ‘Financial Disclosure’ sections, and we ensure that the grant numbers are correct, and we have added it in the cover letter.

6. Thank you for stating the following in your Competing Interests section: 
'The authors declare no conflicts of interest."

Please complete your Competing Interests on the online submission form to state any Competing Interests. If you have no competing interests, please state "The authors have declared that no competing interests exist.", as detailed online in our guide for authors at <http://journals.plos.org/plosone/s/submit-now>
This information should be included in your cover letter; we will change the online submission form on your behalf.

We have completed our Competing Interests on the online submission form and cover letter.

7. PLOS ONE now requires that authors provide the original uncropped and unadjusted images underlying all blot or gel results reported in a submission’s figures or Supporting Information files. This policy and the journal’s other requirements for blot/gel reporting and figure preparation are described in detail at <https://journals.plos.org/plosone/s/figures#loc-blot-and-gel-reporting-requirements> and <https://journals.plos.org/plosone/s/figures#loc-preparing-figures-from-image-files.> When you submit your revised manuscript, please ensure that your figures adhere fully to these guidelines and provide the original underlying images for all blot or gel data reported in your submission. See the following link for instructions on providing the original image data: <https://journals.plos.org/plosone/s/figures#loc-original-images-for-blots-and-gels.>

In your cover letter, please note whether your blot/gel image data are in Supporting Information or posted at a public data repository, provide the repository URL if relevant, and provide specific details as to which raw blot/gel images, if any, are not available. Email us at [plosone@plos.org](mailto:plosone@plos.org) if you have any questions.

We have uploaded the original uncropped and unadjusted images underlying all blot results reported in a submission’s figures as “S1_raw_images”.

**Response to Reviewer 1**

**Thank you for your careful reading and giving us useful suggestions.**

The manuscript entitled “Transcriptome-wide analysis of the difference between MCF7 cells 2 cultured in DMEM or αMEM” aims to demonstrate the effect of culture medium on MCF7 cells in terms of transcriptomic profiling. The manuscript is poorly written and lack the significance and novelty of the study.

We have supplemented the experiments based on the comments from reviewers and made revisions to our manuscript to highlight the key points. We hope this manuscript is appropriate for publication.

1. What is the rationale of this study? Cells grown in different culture media will definitely give different phenotype. Every cell culture has its definite and defined media to culture and proceed the analysis.

Although it is well known that different culture media can cause different phenotypes in cells, we do not yet know in which specific aspects they impact on, especially on the cellular genome. Also, few people would spend time and experience doing this study. Therefore, our research can provide a reference.

2. Pease provide the details of RNA sequencing, like library preparation and sequencing. Please provide PCA for the QC of RNA sequencing.

We have added these contents to “RNA isolation and RNA-Seq library preparation” and “Differential gene expression and gene enrichment analysis” of “Materials and Methods” in manuscript.

3. English mistakes are prevalent throughout the manuscript. Please rectify.

Thank you for your reminder, and we have rectified these mistakes.

4. Missing legends of the figures. Hard to assess the figures and correlate with the results.

We have added the legends of the figures.

5. The manuscript lacks mechanism.

We will further study the mechanism in the future.

**Response to Reviewer 2**

**Thank you for your careful reading and giving us useful suggestions.**

The authors have examined the effect of two different types of cell culture media on breast cancer model 'MCF7'. Based on RNA sequencing data they found that culturing the MCF7 cells in different culture media led to differential gene expression. Moreover, they found that cells that were cultured in alpha-MEM were hypoxic. This study reveals that culture media plays an important role on cell growth property at morphological, biological and transcriptional level, thus it is utmost important to choose the correct cell culture media for a specific cell type to obtain reliable results. The present study reveals interesting findings; however, there are several essential points that need to be addressed before publication.

Minor Comments:

1) In Introduction line no 75, authors have mentioned about NP cells without its introduction. Please explain a little bit more about NP cells and its significance here in this current study.

Thank you for your reminder. We have carefully read the context and found that NP cells are indeed abrupt and unimportant here in this current study, so we have deleted this sentence.

2) It would be interesting if authors could also show the effect of different culture media on cell growth and proliferation by colony forming assay.

We have performed cell colony forming experiments and included the results in the manuscript.

3) In all the results (graphs) please include individual data points that would give a clear indication about number of replicates in each group.

We have remade the graphs to include individual data points.

4) What was the effect of different cultures media on cell attachment to the surface of the cell culture flasks. Were there any differences observed by authors in terms of cell attachment.

We have performed cell attachment experiments and included the results in the manuscript.

5) Did authors find any differential effect of culturing MCF7 in Mitochondrial respiration and intracellular ATP levels or morphological changes at organelle level?

We have detected the intracellular ATP levels and included the results in the manuscript.

6) It would be interesting to look at the effect of culturing MCF7 in different culture media on the expression of MCF7 specific genes like Insulin like growth factor binding protein 2, WNT7B and LHR expression?

We conducted t-tests on the expression values (FPKM) of three genes. The results were that the p-value of the Insulin like growth factor binding protein 2 gene was 0.01977, which was slightly higher in C1, and the p-value of WNT7B and LHR were 0.05227 and 0.9694, which were no significant difference.
